# Endosialin Expression in Metastatic Melanoma Tumor Microenvironment Vasculature: Potential Therapeutic Implications

**DOI:** 10.1007/s12307-015-0168-8

**Published:** 2015-06-18

**Authors:** Eiji Kiyohara, Nicholas Donovan, Ling Takeshima, Sharon Huang, James S. Wilmott, Richard A. Scolyer, Peter Jones, Elizabeth B. Somers, Daniel J. O’Shannessy, Dave S. B. Hoon

**Affiliations:** Department of Molecular Oncology, John Wayne Cancer Institute, 2200 Santa Monica Blvd, Santa Monica, CA USA; Department of Pathology, Melanoma Institute Australia, The University of Sydney and Royal Prince Alfred Hospital, Sydney, NSW 2060 Australia; Biotechnology Science, John Wayne Cancer Institute, 2200 Santa Monica Blvd, Santa Monica, CA USA; Translational Medicine and Diagnostics Morphotek, Inc., 210 Welsh Pool Road, Exton, PA USA; Department of Molecular Oncology, John Wayne Cancer Institute at Providence Saint John’s Health Center, 2200 Santa Monica Blvd, Santa Monica, CA 90404 USA

**Keywords:** Melanoma, Endosialin, CD248, TEM-1, MAb 9G5

## Abstract

Ontuxizumab (MORAb-004) is a humanized recombinant antibody targeting endosialin (TEM-1, CD248). We conducted an analysis of endosialin expression in metastatic melanoma specimens using the anti-endosialin rat anti- MAb 9G5, in order to determine the potential of endosialin as a therapeutic target within the tumor microenvironment vasculature. Endosialin expression in paraffin-embedded archival tissue block (PEAT) melanoma tissues was assessed using immunohistochemistry (IHC) with the anti-endosialin, MAb 9G5, in the vessels of American Joint Commission on Cancer (AJCC) Stage III (*n* = 18) and Stage IV (*n* = 48) specimens. IHC for endosialin expression was further performed on a TMA that included 136 Stage IV and 33 paired Stage III melanoma specimens. BRAF mutation (mt) was also evaluated in individual melanoma specimens and as well as the TMA. Analysis showed 70 % of melanoma specimens (*n* = 46) were positive for endosialin expression. There was no significant difference in endosialin and BRAFmt expression between stages III vs. IV specimens. Endosialin expression was detected in 86 % (*n* = 117) of stage IV TMA specimens, while no expression was detected in 29 normal tissue controls. MAb 9G5 detects the presence of endosialin in the microenvironment tumor vasculature of most metastatic melanoma tissues, regardless of clinical stage and presence of BRAFmt. Endosialin may be a potential therapeutic target by virtue of its selective expression in metastatic melanoma relative to normal tissues.

**Electronic supplementary material:**

The online version of this article (doi:10.1007/s12307-015-0168-8) contains supplementary material, which is available to authorized users.

## Introduction

Ontuxizumab (MORAb-004) is a humanized recombinant mouse antibody (Ab) targeting endosialin (TEM-1, CD248) [1]. The mature form of endosialin is a 175 kDa O-glycosylated transmembrane protein, with an extracellular region characterized by the presence of a carbohydrate-binding lectin like domain and epidermal growth factor (EGF)-like repeats [2, 3]. Endosialin is predominantly expressed in tumor blood vessels [4]. High expression of endosialin has been observed in several solid tumors including colorectal cancer [5], glioblastoma [6], and breast cancer [7]. In metastatic melanoma, endosialin is selectively expressed in subsets of small- and medium-sized tumor vessels [8].

Endosialin expression has previously been detected in tumor pericytes and stromal cells by immunohistochemistry (IHC) [9]. Intratumoral pericytes and stromal cells play an important role in tumor progression and metastasis. Tumor-associated fibroblasts in the stroma contribute to migration and invasion by producing extracellular matrix proteins, matrix metalloproteinases (MMPs) and growth factors [10, 11]. These factors can recruit endothelial precursor cells that initiate angiogenesis. Platelet-derived growth factor-B (PDGF-B), which is secreted from cancer cells, recruits pericytes that express PDGF receptor-β (PDGFR-β) on tumor vessels [12, 13]. In endosialin overexpressed transgenic mice, the pericyte marker, neurite growth proteoglycan 2 (NG2), is co-expressed with endosialin in tumor vessels but not in CD31-positive endothelial cells [14]. Thus, endosialin seems to be primarily expressed on pericytes as opposed to endothelial cells in the tumor vasculature.

In endosialin-null mice, abdominal implanted tumors have reduced tumor growth, invasion, and metastasis when compared to wild type mice [15]. Tomkowicz, B., et al. [16] reported that endosialin regulates proliferation of pericytes through a PDGFR signaling pathway via mitogen-activated protein (MAP) kinase/extracellular signal-regulated kinase 1/2 (ERK1/2). This data suggests that the suppression of endosialin in the tumor vasculature may lead to a reduced number of pericytes, which contributes to the prevention of tumor progression.

Treatment of aggressive metastatic melanoma has been a daunting challenge. Several chemotherapies have been utilized for first-line treatment, but very few durable responses are achieved. Recently, novel molecular-targeted therapies have been approved by the FDA for first-line treatment in advanced melanoma i.e., vemurafenib for BRAF V600E mutation (BRAFmt) [17]. However, patients receiving anti-BRAFmt drugs often experience recurrence and develop acquired resistance within 1 year. Most recently, programmed cell death protein 1(PD-1) inhibitor has shown significant clinical activity and has been approved for use in melanoma patients. Nonetheless, most patients either do not respond or have a likelihood of recurrence, thus there remains a great clinical need to identify new drug targets and treatment strategies.

This study is the first large survey of endosialin protein expression in clinical melanoma specimens at different disease stages. Our data shows that endosialin is expressed in most metastatic melanoma (70-86 %) and not in the normal tissue, supporting it as a potential new therapeutic target for advanced stage melanomas. The aim of this study was to determine endosialin expression in melanomas as a prelude to in vivo testing and clinical trials, which will eventually determine the potential of endosialin targeting for patients with metastatic disease.

A rat MAb anti-endosialin (9G5) was assessed in cutaneous melanoma tissue specimens. The MAB 9G5 is very specific for endosialin/TEM-1/CD248. The MAb specifically stains the tumor vasculature and stromal regions of the tumor microenvironment. TEM-1 expression on pericytes and stromal fibroblast of tumor is significantly upregulated and stained by MAb 9G5. Furthermore, the membranes of perivascular, normal and non-vascular stroma, as well as tumor and endothelial cells rarely stain for TEM-1.

## Materials and Methods

### Paraffin-Embedded Archival Tissue

Melanoma patients treated at Saint John’s Health Center between 1995 and 2011 were reviewed for inclusion in this study. Approval for the use of human tissues was obtained from the Saint John’s Health Center/John Wayne Cancer Institute joint institutional review board and Western institutional review board. Informed consent was obtained from all patients. PEAT (*N* = 66) and frozen tissue (*N* = 16) specimens were obtained from AJCC Stage III and IV melanoma patients who had either regional lymph node (LN) or distant organ metastasis. Clinical data was retrieved from the John Wayne Cancer Institute (JWCI) melanoma database.

### Melanoma Tissue Microarray

TMA data was constructed using AJCC stage IV melanomas from JWCI with annotated clinical outcome as previously described [18]. The melanoma specimens were derived from representative areas of each PEAT block. Cores measuring 0.6 mm in diameter were made for each sample. The AJCC Stage IV melanoma TMA included 268 distant organ metastases and 39 paired AJCC Stage III regional LN metastases from 169 melanoma patients, as well as 29 normal tissue controls from each respective organ (cancer-free). Duplicated cores of all specimens and controls were included in the TMA.

### Immunohistochemistry of PEAT and TMA

Immunohistochemistry (IHC) was performed as previously described using the following reagents and Abs [18]. PEAT specimens were sectioned at 5 μm and fixed on positively-charged glass slides. After deparaffinization and rehydration, the slides were heated for 30 min at 100 °C in Diva heat-induced epitope retrieval solution (Biocare Medical, Concorde, CA). Peroxidase-1 blocking solution (Biocare Medical, Concorde, CA) was applied to specimens and incubated at room temperature for 5 min. Tissue sections were then blocked with 5 % goat serum for 30 min followed by 60 min incubation at room temperature with 1.25 μg/mL (1:1200 dilution) of anti-endosialin clone 9G5 primary rat MAb (Morphotek, Inc.) diluted in Ab Diluent (Dako, Glostrup, Denmark). The slides were then incubated for 30 min with 7.5 μg/ml of biotinylated anti-rat secondary link Ab (Vector Labs, Burlingame, CA) and developed with ABC detection reagent (Vector Labs, Burlingame, CA) and 3’3’ diaminobenzidine (DAB) chromogenic substrate (Dako, Glostrup, Denmark). Tissue sections were counterstained with hematoxylin for 2 min.

### IHC of Frozen Tissue

Frozen tissues of AJCC stage III and IV melanoma patients were prepared with paired PEAT blocks. Frozen tissues were cryosectioned (5 μm) at −20 °C with Cryocut 1800 (Leica, Nussloch, Germany) and adhered to slides using CryoJane® (Instrumedics, Ann Arbor, MI) and adhesive tape window (Leica Microsystems, Wetzlar, Germany). The tissue on the tape was transferred to a positively charged slide. After drying, the slides were stored in a −80 °C freezer for IHC staining. Prior to the staining procedure, stored slides were air-dried at room temperature and then fixed in 3 % formaldehyde for 15 min. Following the TBST wash, the peroxidase-1 blocking and the remaining consecutive steps were followed as described above.

### Analysis of Endosialin Expression

IHC staining for endosialin expression in 66 PEAT blocks and 16 frozen specimens was analyzed using ImageJ software (http://rsbweb.nih.gov/ij). Only vascular regions were marked by ImageJ and the color was inverted in black and white mode. Average intensity of endosialin staining for each marked vascular region was based on the total intensity divided by the area. The results of endosialin expression were obtained from the average of two different regions from each specimen. IHC analysis of endosialin expression in TMA was scored by a dermatologist (E.K.). After endosialin staining, specimens without vascular region were excluded from IHC scoring. Finally, the IHC for endosialin expression in vascular regions was classified as score 0 (absent), score 1 (weak), score 2 (moderate), and score 3 (strong) by light microscopy as previously described [18].

### DNA Extraction

The PEAT blocks for DNA extraction were cut to 10 μm sections, deparaffinized and stained with hematoxylin. One 5 μm section was cut from each tissue and stained with hematoxylin and eosin (H&E) to identify tumor region; thereafter, a 26G needle was used to isolate tumor tissue for DNA extraction and then subjected to Proteinase K digestion at 50 °C for 16 h. . ZR FFPE DNA MiniPrep^TM^ Kit (Zymo Research, Irvine, CA) was then utilized for DNA extraction in accordance with the manufacturer’s instructions. DNA contaminated with melanin was further purified by OneStep™ PCR Inhibitor Removal Kit (Zymo Research, Irvine, CA).

### Analysis of BRAF V600E Mutation

BRAF (V600E) status for each PEAT specimen was assessed by TaqMan mutation detection assays competitive allele-specific PCR (Applied Biosystems, Foster City, CA). TaqMan PCR were run in 384 wells plate including 5 μl of 2X TaqMan® genotyping master mix (Applied Biosystems), 0.5 μl of 10X assay mix for allele 1 (or 2), 2.5 μl of deionized water and 2 μl of DNA template (40 ng) according to the manufacturer’s instruction. PCR were performed in duplicate on the ABI Prism 7900 HT sequence detection system (Applied Biosystems) using the following thermo cycling conditions: 95 °C for 10 min; 5 cycles of 92 °C for 15 s and 58 °C for 1 min; 40 cycles of 92 °C for 15 s; and 60 °C for 1 min. Collected data were then analyzed with the SDS 2.0 software program per manufacturer’s instruction. For each run, proper positive and negative controls were included. BRAFmt of the melanoma TMA was stained with monoclonal mouse antibody VE1 as described previously [19]. Specimens with no tumor, pigmentation or loss of tumor cores were excluded from IHC scoring. Finally, IHC for BRAFmt detection was classified as score 0 (negative) or score 1 (positive) by light microscopy.

## Results

### MAb 9G5 Detected Endosialin Expression in Metastatic Melanomas

**Fig. 1:**
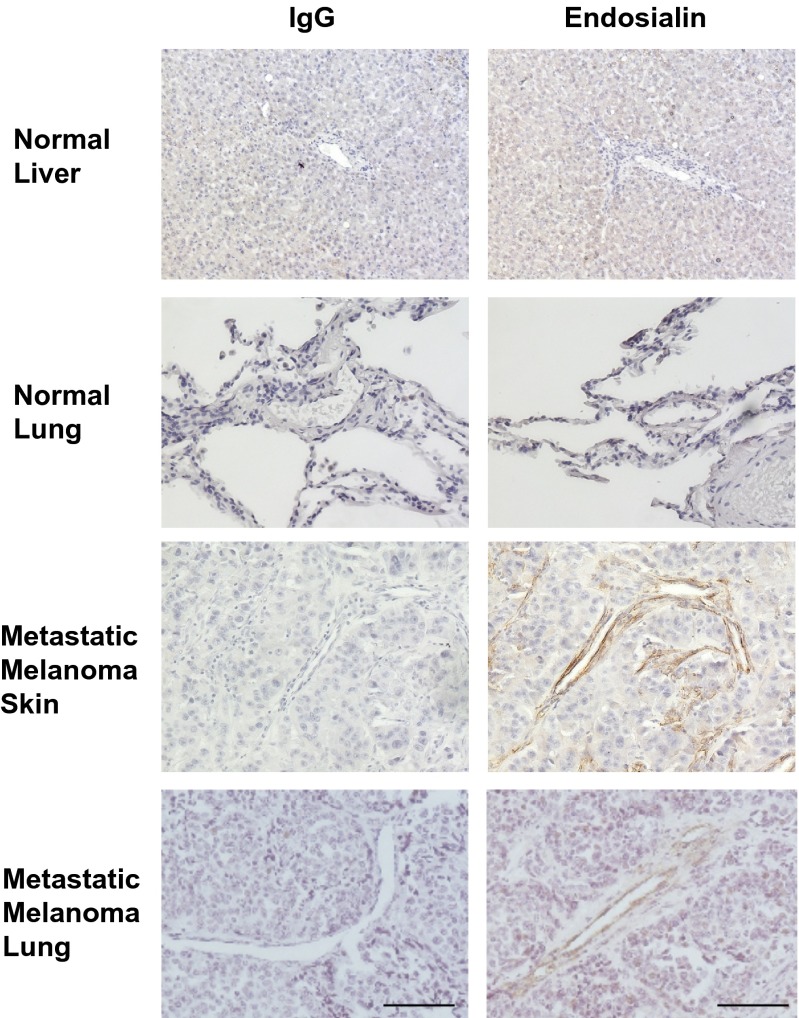
Representative photos of endosialin expression in melanoma and normal tissues IHC was performed on normal liver, lung and metastatic melanoma tissues with MAb 9G5. Normal tissues revealed no endosialin expression. The vascular regions of metastatic melanoma showed strong endosialin expression. IgG: negative control staining with isotype Ab and endosialin: TEM-1 staining with MAb 9G5. Scale bar, 100 μm

To evaluate the specificity and sensitivity of IHC methodology in detecting endosialin expression in the tumor microenvironment vasculature of metastatic melanoma using MAb 9G5, we compared endosialin expression in melanoma PEAT specimens with that of two normal tissue types (liver and lung) as controls (Fig. 1). Endosialin expression was significantly up-regulated within tumor vessels and stromal region of the melanoma specimens, while none was observed in the vascular regions of the normal controls. Moreover, as shown in a previous report, hepatocytes exhibited weak expression of endosialin, [20].

### Correlation of Endosialin Expression with BRAFmt

Approximately 40–60 % of melanoma patient’s tumors reportedly harbor the BRAFmt [21, 22]. We analyzed the correlation of endosialin expression with clinical stage and BRAFmt status of 25 PEAT specimens from AJCC stage III and IV (8 and 17, respectively). Endosialin expression was positive in 75 % (*n* = 6) of AJCC Stage III and 41 % (*n* = 7) of AJCC Stage IV specimens, with an overall expression rate of 52 % (Table 1a). BRAFmt was detected in 38 % (*n* = 3) of AJCC Stage III and 35 % (*n* = 6) of AJCC Stage IV melanoma specimens (Table 1b). Furthermore, endosialin expression was detected in 33 % (*n* = 3) of BRAFmt positive and 63 % (*n* = 10) of BRAFmt negative specimens (Table 1c). The apparent trend toward decreased endosialin expression in BRAFmt positive specimens was not statistically significant; this trend was reversed in a TMA analysis (see below); therefore we detected no consistent correlation of endosialin expression with BRAFmt status. Endosialin was expressed in the melanoma vasculature, regardless of BRAFmt.

**Table 1:** Endosialin expression associated with BRAFmt

A			
Endosialin	AJCC Stage III (%)	AJCC Stage IV (%)	Total (%)
+	6 (75)	7 (41)	13 (52)
–	2 (25)	10 (59)	12 (48)
Total	8 (100)	17 (100)	25 (100)
B			
BRAFmt	AJCC Stage III (%)	AJCC Stage IV (%)	Total (%)
+	3 (38)	6 (35)	9 (36)
–	5 (72)	11 (65)	16 (64)
Total	8 (100)	17 (100)	25 (100)
C			
Endosialin	BRAFmt–(%)	BRAFmt + (%)	Total (%)
+	10 (63)	3 (33)	13 (52)
–	6 (27)	6 (67)	12 (48)
Total	16 (100)	9 (100)	25 (100)

### Endosialin Expression was Similarly Detected in PEAT and Frozen Melanomas

We analyzed 16 paired PEAT and frozen tissues (AJCC Stage III and IV) to assess epitope retention, following formalin fixation (Supplemental Figure 1A). Endosialin expression of PEAT melanoma was detected in 80 % (*n* = 4) of stage III and 82 % (*n* = 9) of stage IV specimens (Supplemental Table 1A and Supplemental Figure 1B). Similarly, endosialin expression of frozen tissues was detected in 80 % (*n* = 4) of stage III and 73 % (*n* = 8) of stage IV melanoma specimens (Supplemental Table 1B, Supplemental Figure 1C). Consequently, no significant difference in endosialin expression between PEAT and frozen metastatic melanoma tissues was observed (Supplemental Figure 1D), demonstrating the efficiency of using MAb 9G5 to detect endosialin in both PEAT and frozen melanoma specimens.

### Exclusion of Pre-treatment Effect on Endosialin Expression

There is a possibility that chemotherapy pre-treatment, such as dacarbazine (DTIC), which patients received before tumor resection, may have affected endosialin expression. To exclude this possibility, an additional 25 PEAT specimens from stage III and IV (5 and 20, respectively) patients were analyzed. This cohort was restricted to patients who did not receive any treatment 2 months prior to an operation. Endosialin positive expression was detected in 80 % (*n* = 4) of stage III and 80 % (*n* = 16) of stage IV specimens (Table 2), similar to the previous set of PEAT melanoma specimens. Therefore, these results suggest that chemotherapy pre-treatment did not affect the level of endosialin expression in the previously analyzed treated melanoma PEAT specimens.

**Table 2:** Endosialin expression in melanoma tissues among patients without pre-surgery treatment

Endosialin	AJCC Stage III (%)	AJCC Stage IV (%)	Total (%)
+	4 (80)	16 (80)	20 (80)
–	1 (20)	4 (20)	5 (20)
Total	5 (100)	20 (100)	25 (100)

### Comparison in Endosialin Expression Detected Between Stage III and Stage IV Specimens

**Fig. 2:**
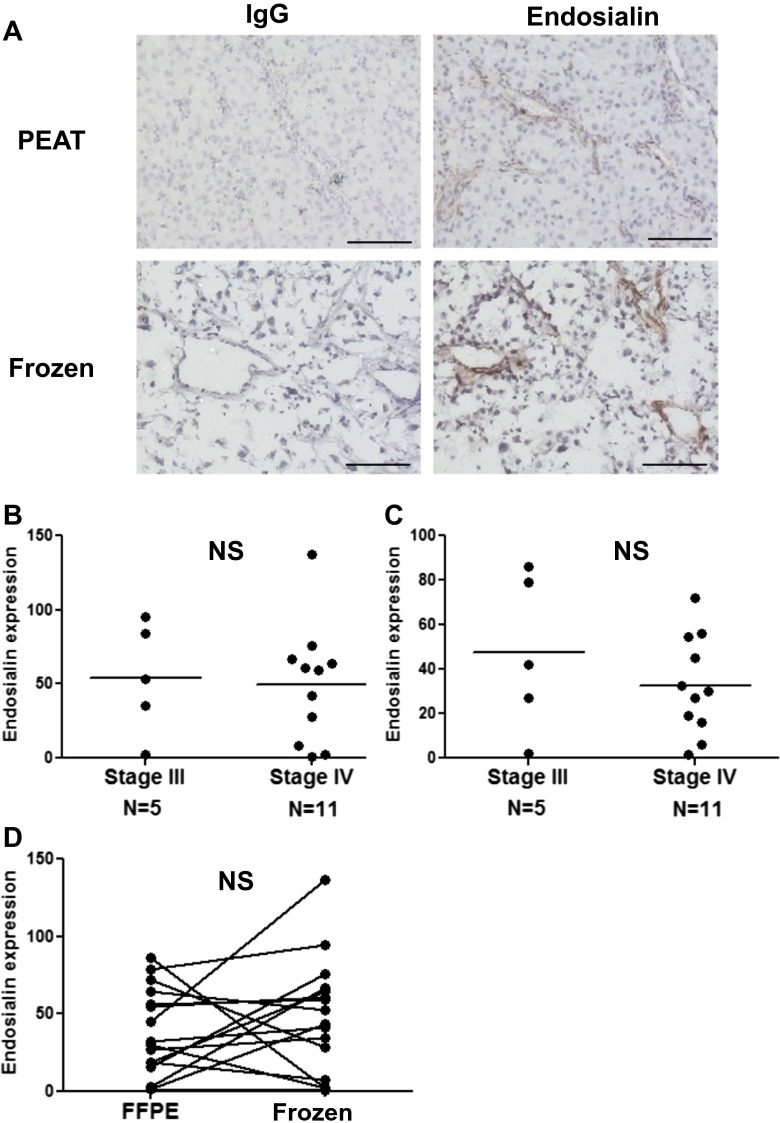
Endosialin expression in paired PEAT and frozen OCT melanoma specimens **a**. Endosialin IHC staining of metastatic gallbladder PEAT specimens and OCT specimens. IgG: negative control staining with isotype Ab and endosialin: Endosialin staining with MAb 9G5. Scale bar, 100 μm. **b**. Comparing endosialin expression levels between stage III and stage IV PEAT melanoma specimens. **C**. Comparing endosialin expression between stage III and stage IV OCT frozen melanoma specimens. **d**. Comparing endosialin expression between PEAT melanoma specimens and OCT melanoma specimens. Statistical analysis was conducted by Fisher’s exact test in B and C. NS, not significant

**Fig. 3:**
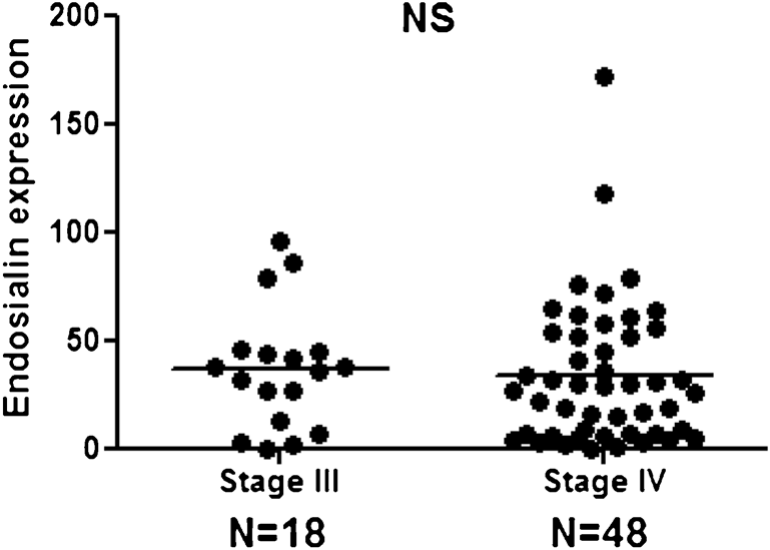
Endosialin expression in AJCC Stage III and IV Melanomas by IHC analysis Endosialin expression levels for PEAT AJCC Stage III and Stage IV melanoma specimens with MAb 9G5 IHC staining. Statistical analysis was assessed by Fisher’s exact test. NS, not significant

All the PEAT melanoma specimens assessed for endosialin expression with IHC (18 stage III and 48 stage IV melanoma patients) were compared by stage. 78 % (*n* = 14) of stage III and 67 % (*n* = 32) of stage IV showed positive expression of endosialin (Fig. 2 and Table 3). There was no significant difference in the frequency of endosialin expression between stage III and IV metastatic melanomas in this cohort. This suggested that the expression of endosialin is not dependent on regional or distal disease status (Fig. 3).

**Table 3:** Endosialin expression of stage III and IV melanomas

Endosialin	AJCC Stage III (%)	AJCC Stage IV (%)	Total (%)
+	14 (78)	32 (67)	46 (70)
–	4 (22)	16 (23)	20 (30)
Total	18 (100)	48 (100)	66 (100)

### TMA Data Analysis

We analyzed endosialin expression in the vasculature of melanoma with a well annotated Stage III and IV melanoma TMA. We excluded cores that lacked vasculature from the TMA analysis, resulting in a cohort of 136 Stage IV and 33 paired Stage III melanoma specimens obtained from patients with metachronous metastases, as well as 29 normal tissue controls. Endosialin expression with IHC scores of 1, 2 or 3 were detected in 86 % of Stage IV and 82 % of Stage III melanoma specimens (Table 4) i.e., similar to the data obtained on examination of whole tumor PEAT sections. In addition, no endosialin expression was detected in the normal tissue controls. Table 5 shows the metastatic sites for patients, along with endosialin expression. Gender, ulceration, and metastatic site showed no correlation with endosialin expression. BRAFmt status, as previously stated, also showed no significant correlation with endosialin expression in the TMA analysis (Table 6). However, endosialin expression was observed in 92 % of specimens with the BRAFmt.

**Table 4:** Clinicopathological factors of TMA data

Endosialin	AJCC Stage III (%)	AJCC Stage IV (%)	Total (%)
+	27 (82)	117 (86)	144 (85)
–	6 (18)	19 (14)	25 (15)
Total	33 (100)	136 (100)	169 (100)

**Table 5:** ᅟ

	Endosialin			
Organ	Negative	Positive	Total	% Positive
Adrenal gland	1	7	8	88
Bone	0	6	6	100
Bowel	5	25	30	83
Skin	1	21	22	95
Liver	2	4	6	67
Lung	5	28	33	85
Muscle	2	5	7	71
Pancreas	1	5	6	83
Brain	1	2	3	67
Gallbladder	1	1	2	50
Kidney	0	2	2	100
Ovary	0	2	2	100
Peritoneum	0	1	1	100
Soft tissue	0	3	3	100
Spleen	0	4	4	100
Gastric	0	1	1	100

**Table 6:** ᅟ

Endosialin	BRAFmt–(%)	BRAFmt + (%)	Total (%)
+	51 (84)	35 (92)	86 (87)
–	10 (16)	3 (8)	13 (13)
Total	61 (100)	38 (100)	99 (100)

## Discussion

Angiogenesis is important for tumor growth, invasion and metastases of cancers. Therefore, treatments targeting angiogenesis have been used for several cancer types with variable clinical response [23]. Endosialin may represent an angiogenesis related target for melanoma.

Vascular endothelial growth factor (VEGF), which induces angiogenesis and plays a critical role in the regulation of vasculogenesis, is an important therapeutic target.[24] Receptor tyrosine kinase (RTK) inhibitors have also been developed, which block c-KIT and PDGFR [25]. One of the RTK inhibitors, Sunitinib, also inhibits VEGFR [25]. Pericytes expressing PDGFR and endothelial cells expressing VEGFR are thought to be the targets of anti-angiogenesis treatments. While a therapeutic strategy targeting endosialin may also prove effective, the role of pericyte endosialin in cancer remains unclear. A recent study showed that endosialin on pericytes binds to the capillary basement membrane and promotes endothelial cell apoptosis [26]. Cell-cell interactions between pericytes and endothelial cells appear to be required for vessel stabilization. Taken together, these data suggest that pericytes play a crucial role in promoting vessel maturation along with selective vessel destabilization and regression. Therefore, blocking endosialin function may prevent tumor vasculature remodeling, which may ultimately diminish tumor blood flow.

The evidence supporting the therapeutic potential of inhibiting endosialin is conflicting. In support of endosialin growth enhancing effects, endosialin signaling shares ERK-1/2 with PDGFR-β signaling to increase pericytes [16]. Inhibition of PDGFR-β eliminated PDGFR-β^+^ progenitor perivascular cells and pericyte quantity [27]. Overexpression of PDGF-BB (PDGF-B homodimer) in B16F10 melanoma cells increased the proliferation of pericytes and tumor growth [28] and overexpression of endosialin enhanced cell migration in Chinese hamster ovary cells [29]. Growth of colon tumors implanted in the liver and large intestine was impaired in endosialin ^−/−^ mice [15]. In transgenic mice, deletion of the cytoplasmic domain of endosialin suppressed tumor growth, suggesting a role for this domain in signal transduction [30]. Furthermore, elevated endosialin expression (analyzed by an RT-PCR) in breast tumors has been associated with poor prognosis [7]. Taken together, these studies imply that endosialin has a role in promoting tumor growth.

Conversely, overexpression of PDGF-BB in colorectal and pancreatic cancer cell lines suppressed tumor growth and was associated with increased pericytes in subcutaneous and orthotopic tumors [31]. In an endosialin KO model, tumor size was not decreased in a subcutaneous colon tumor [15] or intracranial glioblastoma multiform [32]. Thus, the effect of endosialin inhibition is not assured and in vivo studies followed by clinical trials will be needed to establish MORAb-004’s therapeutic potential.

While the BRAFmt is the most frequently detected mutation in melanoma, in our study we found no correlation of the presence of this mutation with endosialin expression, and endosialin is expressed in 92 % of TMA specimens with the presence of BRAFmt. Therefore, the presence or absence of BRAFmt may not influence the clinical response to MORAb-004, and this study would not support patient selection on that basis. However, whether the expression of endosialin is upregulated in BRAF inhibitor –resistant melanoma is interesting and remains to be determined.

In conclusion, we have demonstrated that endosialin can be recognized by the MAb 9G5 antibody in both PEAT melanoma and frozen tissues by IHC analysis. In addition, we showed that endosialin is highly expressed in the tumor vasculature of metastatic melanoma tissues, regardless of clinical stage and BRAFmt status, and that endosialin is expressed in most BRAFmt melanomas in a TMA. Although the effect of inhibiting endosialin function cannot be predicted with certainty prior to the outcome of human clinical trials, the findings support further investigation of endosialin as a potential therapeutic target in melanoma using the MORAb-004 antibody. This study supports further in vivo and clinical investigations to establish the MORAb-004 antibody’s potential for melanoma treatment. This study is highly important in that targeted antigen MAB therapies need a reliable surrogate biomarker pathology assay to verify the target antigen present and level in the tumor or tumor microenvironment as in this study. More interestingly, endosialin is in the tumor microenvironment vasculature where it may be a more effective target since heterogeneity may be more stable than in the tumor [33]. Both PEAT and frozen tissues were shown to be assessable for anti-endosialin MAB.

## Electronic supplementary material

ESM 1 (DOCX 20 kb)

ESM 2 (PPTX 313 kb)

## Abbreviations

(AJCC): American Joint Committee on Cancer
(Ab): antibody
(BRAFmt): BRAF V600E mutation
(DAB): diaminobenzidine
(EGF): epidermal growth factor
(ERK1/2): extracellular signal-regulated kinase 1/2
(H&E): Hematoxylin and eosin
(IHC): Immunohistochemistry
(LN): Lymph node
(MMPs): Matrix metalloproteinases
(mt): Mutation
(MAP): Mitogen-activated protein kinase
(NG2): Neurite growth proteoglycan 2
(MORAb-004): Ontuxizumab
(PEAT): Paraffin-embedded archival tissue block
(PDGF-B): Platelet-derived growth factor-B
PDGF: PDGF-BB (PDGF-B homodimer)
(PDGFR-β): Receptor-β
1(PD-1): Programmed cell death protein
(RTK): Receptor tyrosine kinase
(TMA): Tissue microarray
(VEGF): Vascular endothelial growth factor
