# Supplementary material for: Endosialin Expression in Metastatic Melanoma Tumor Microenvironment Vasculature: Potential Therapeutic Implications
Source: Cancer Microenviron. 2015 Jun 18;8(2):111–8. doi: 10.1007/s12307-015-0168-8 (PMC4542822; doi:10.1007/s12307-015-0168-8)
Supplement: Supplementary file 1 — (DOCX 20 kb) [file 12307_2015_168_MOESM1_ESM.docx]

**Supplemental Table 1: Endosialin expression in PEAT and Frozen melanoma specimens**


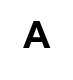


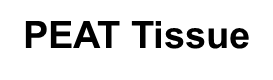


| **Endosialin** | **AJCC Stage III (%)** | **AJCC Stage IV (%)** | **Total (%)** |
| --- | --- | --- | --- |
| + | 4 (80) | 9 (82) | 13 (81) |
| - | 1 (20) | 2 (18) | 3 (19) |
| Total | 5 (100) | 11 (100) | 16 (100) |


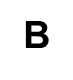


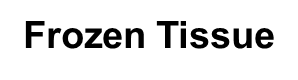


| **Endosialin** | **AJCC Stage III (%)** | **AJCC Stage IV (%)** | **Total (%)** |
| --- | --- | --- | --- |
| + | 4 (80) | 8 (73) | 12 (75) |
| - | 1 (20) | 3 (27) | 4 (25) |
| Total | 5 (100) | 11 (100) | 16 (100) |
