# Supplementary figures and images for: Endosialin Expression in Metastatic Melanoma Tumor Microenvironment Vasculature: Potential Therapeutic Implications
Source: Cancer Microenviron. 2015 Jun 18;8(2):111–8. doi: 10.1007/s12307-015-0168-8 (PMC4542822; doi:10.1007/s12307-015-0168-8)

## Slide 1
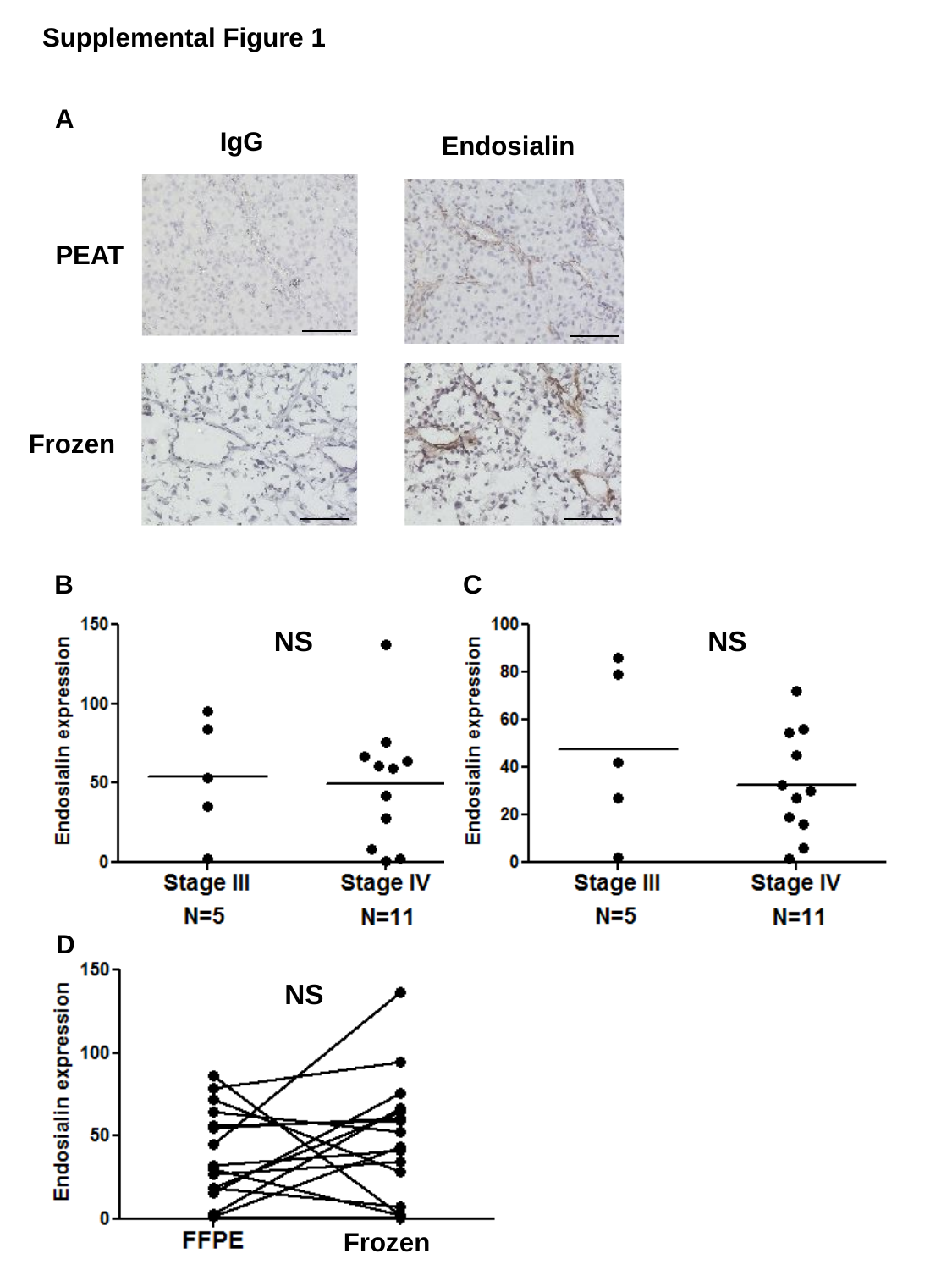

Supplemental Figure 1
A
IgG
Endosialin
PEAT
Frozen
B
C
NS
NS
D
NS
Frozen

Supplement: Supplementary file 2 — (PPTX 313 kb) [file 12307_2015_168_MOESM2_ESM.pptx]
